# Study protocol for daiobotanpito combined with antibiotic therapy for treatment of acute diverticulitis: a study protocol for a randomized controlled trial

**DOI:** 10.1186/s13063-020-04370-7

**Published:** 2020-06-16

**Authors:** Keiko Ogawa-Ochiai, Kenichi Yoshimura, Akiko Shirai, Seisho Sakai, Hideki Moriyama, Keishi Nakamura, Toshinori Murayama, Hideki Ishikawa

**Affiliations:** 1 Department of Japanese Traditional (Kampo) Medicine, Kanazawa University Hospital, 13-1 Takaramachi, Kanazawa City, Ishikawa 920-8641 Japan; 2 Center for Integrated Medical Research, Hiroshima University Hospital, Hiroshima University, Kasumi 1-2-3, Minami-ku, Hiroshima, 734-8551 Japan; 3 Department of Pediatric Surgery, Kanazawa University Hospital, Takaramachi 13-1, Kanazawa, Ishikawa 920-8641 Japan; 4 Department of Thoracic, Cardiovascular and General Surgery, Kanazawa University Hospital, Takaramachi 13-1, Kanazawa, Ishikawa 920-8641 Japan; 5 Department of Gastrointestinal Surgery, Kanazawa University Hospital, Takaramachi 13-1, Kanazawa, Ishikawa 920-8641 Japan; 6 Innovative Clinical Research Center, Kanazawa University Hospital, Takaramachi 13-1, Kanazawa, Ishikawa 920-8641 Japan; 7 Department of Molecular-Targeting Cancer Prevention, Graduate School of Medical Science, Kyoto Prefectural University of Medicine, 3-2-17-2F Imabashi, Chuo-ku, Osaka, 541-0042 Japan

**Keywords:** Japanese traditional (Kampo) medicine, Daiobotanpito, Diverticulitis, Da huang mu dan tang, Randomized controlled trial

## Abstract

**Background:**

Colonic diverticular disease has been increasing in prevalence due to the rapidly aging global population, but standard treatment has not changed dramatically in recent years. Daiobotanpito (DBT; *Da Huang Mu Dan Tang* in Chinese) has been used in medical treatment of acute abdominal abscesses, such as appendicitis or diverticulitis in traditional Japanese (Kampo) medicine for many years, based on more than 3000 years of experience. Prior to this study, a retrospective open-label trial was conducted to compare patients with acute diverticulitis who received oral DBT combined with intravenous antibiotics with those who received intravenous antibiotic alone; it showed a positive effect of DBT on acute diverticulitis. We aim to investigate whether moderate to severe acute diverticulitis shows greater improvement with intravenous antibiotics plus orally administered DBT compared with intravenous antibiotics plus placebo.

**Methods:**

This is a two-group, randomized, double-blind, placebo-controlled, multi-center trial, which is designed to evaluate the efficacy and safety of DBT in patients with moderate to severe diverticulitis treated with intravenous antibiotics. Eligible participants will be randomized to either a treatment group receiving a 10-day oral DBT regimen plus conventional therapy or a control group receiving a 10-day placebo regimen plus conventional therapy. The primary outcome will be success in treating diverticulitis: the success rate will be defined as elimination of abdominal pain within 4 days in all patients, and in patients with fever (body temperature ≧ 37.5 °C) on inclusion into this study, fever relief with reduction in body temperature to < 37.5 °C within 3 days. Secondary endpoints will include the number of hospitalization days, changes in inflammatory response (C-reactive protein (CRP), white blood cell (WBC) and neutrophil counts), fever type, number of days before beginning food intake, recurrence rate (observation for 1 year after registration), and adverse event expression rate. Assessments will be performed at baseline and on the day of discharge. The recurrence rate will be recorded at 1 year after registration.

**Discussion:**

This study is expected to provide evidence to support the clinical benefits of DBT in the treatment of acute diverticulitis. It may also provide evidence on the efficacy and safety of DBT in the recurrence of acute diverticulitis.

**Trial registration:**

UMIN-CTR: UMIN000027381. Registered on 27 April 2017.

https://upload.umin.ac.jp/cgi-bin/ctr/ctr_view_reg.cgi?recptno=R000031377, and changed to jRCTs041180063, registered on 30 July 2019; as a result of the revision of the domestic law in 2018 in Japan.

## Background

The prevalence of diverticular disease is estimated to range between 20% and 60% in the general population worldwide [1, 2]. Although 75% of patients with diverticulosis remain symptom-free during their lifetime, the prevalence of patients who require medical or surgical treatment has increased by 16% in the last 20 years, thereby increasing morbidity associated with this condition [3–5]. According to the recommendations from the American Gastroenterological Association on treatment and diagnosis [6] and the Guidelines for Colonic Diverticulitis of the Japan Gastroenterological Association [7], the initial treatment of uncomplicated colonic acute diverticulitis is bowel rest (i.e., fasting) and broad-spectrum antibiotics. Notably, despite the presence of abscess or perforation with colonic diverticulitis, conservative treatment is typically performed for limited peritonitis and abscess. Patients who do not respond to medical therapy should be considered for surgery depending on the clinical situation. Elective resection is recommended after two well-documented attacks, depending on the patient’s age and medical fitness and the severity of the attack.

Moreover, colonic diverticular disease has been increasing in prevalence due to the rapidly aging global population [6]. However, standard treatment has not changed noticeably in recent years.

The unique application of traditional Japanese (Kampo) medicine is gradually attracting worldwide attention. In Kampo medicine, daiobotanpito (DBT; *Da-Huang-Mu-Dan-Tang*) is used for patients with *Yang* (*Yang qi* is a kind of *qi* that mainly has a warming function) - excess in the interior layers of the body, who exhibit signs of local *Qi* (a kind of vital energy) - congestion and blood stagnation. It has traditionally been used to treat abscesses in the intestine, such as in diverticulitis or appendicitis. From the perspective of Kampo medicine, DBT drains heat, dispels blood stasis, disperses clumping, and reduces swelling. It is primarily used in patients with abdominal distention and constipation. DBT consists of five crude drugs: *Rhei rhizoma*, *Natrium sulfuricum*, *Moutan cortex*, *Persicae semen*, and *Benincaseae semen*. Notably, *Rhei radix et Rhizoma* and *Moutan cortex* drain heat and dispel blood stasis. These aspects are attributed to heat and constraints that allow the formation of toxins, which secondarily leads to the formation of pus. The appropriate strategy in Kampo medicine is to discharge the toxin, eliminate the phlegm (the viscous turbid pathological product that can accumulate in the body, causing a variety of diseases), clear the heat, and clear the blockage [8].

As described previously, surgery should be performed in patients who do not respond to medical therapy in difficult or complicated clinical situations. DBT may be an alternative or supportive therapy for patients with acute diverticulitis, which may enable these patients to avoid surgery. Prior to this study, a retrospective open-label trial was conducted to compare patients with acute diverticulitis who received oral DBT combined with intravenous antibiotics with those who received intravenous antibiotics alone; importantly, it showed a positive effect of DBT for treatment of acute diverticulitis. The study included 34 patients: 11 patients in group 1 receiving DBT and 23 patients in group 2 not receiving DBT. There was a significantly improved outcome in the group treated with DBT, compared to the group not treated with DBT, when comparing duration of fever, abdominal pain, and administration of antibiotics [9].

The authors hypothesize that combined treatment with oral DBT is superior to the standard therapy for acute diverticulitis. This study aims to investigate the effectiveness of the treatment of acute colonic diverticulitis with Kampo medicine, DBT.

## Methods/design

### Study design and settings

**Fig. 1.**
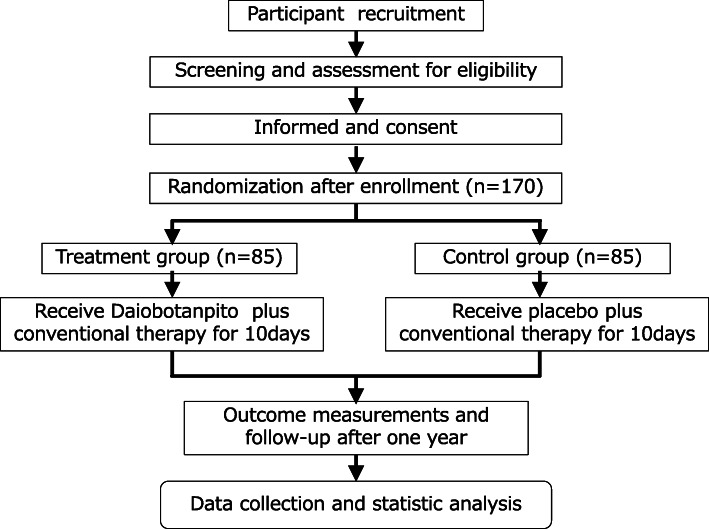
Study design flow chart

A brief flowchart of the entire study is shown in Fig. 1. This study is a two-group, randomized, double-blind, placebo-controlled, multicenter trial, which will evaluate the efficacy and safety of DBT in patients with acute diverticulitis. The study will be conducted throughout Japan. Patients will be recruited from the gastroenterology inpatient departments of 13 hospitals. Informed consent will be obtained from all study participants. The study protocol was designed in accordance with the ethical principles in the Declaration of Helsinki and regional regulations. Central ethical approval of this study has been confirmed from the Central Review Board of Kanazawa University Hospital (reference approval number 6058) and we will not begin recruiting at other centers in the trial until local ethical approval has been obtained. This study was registered at https://upload.umin.ac.jp/cgi-bin/ctr/ctr_view_reg.cgi?recptno=R000031377 (UMIN000027381) on 27 April 2017. We had to change our registration from UMIN to https://jrct.niph.go.jp/en-latest-detail/jRCTs041180063 (jRCTs041180063) on 30 July 2019. In Japan, as a result of the revision of the domestic law in 2018, the clinical trial register in accordance with the domestic law has been officially changed for almost all investigator-initiated clinical trials of new investigational drugs. So, we registered the study again with the ethical committee, which was organized according to the new law. After their approval, we changed the registered website from UMIN to jRCT.

The protocol includes elements recommended in the Standard Protocol Items: Recommendations for Interventional Trials (SPIRIT) [10] checklist (Additional file 1).

### Setting and participants

This study will be conducted in Japan. Patients will be recruited from the gastroenterology and surgery inpatient department at each of the hospitals in different districts in Japan. Participants who meet the following inclusion criteria are eligible:

1. Moderate to severe diverticulitis: diagnosis will be based on computed tomography (CT) evidence of diverticula-like structure, combined with tenderness or abdominal pain, thickening of the colon wall, signs of inflammation of pericolonic fat, tissue density, and vascular involvement. Pericolonic abscess, free air or extravasation, and accumulation of fluid will also be assessed to predict prognosis.
2. Age at least 20 years and younger than 75 years.
3. Ability to communicate with the investigators.
4. Abdominal pain and/or fever with body temperature > 37.5 °C.
5. Provision of written informed consent for participation after receiving sufficient explanation for participation in this study and achieving sufficient understanding.

Participants who meet the following exclusion criteria will not be enrolled:

1. Severe organ dysfunction as follows, based on blood tests within 4 the weeks before treatment starts:
  - Renal function: serum creatinine value 1.5-fold greater than the upper limit of the institutional standard.
  - Liver function: serum aspartate aminotransferase (AST) value or serum alanine aminotransferase (ALT) threefold greater than the upper limit of the institutional standard.
  - Central nervous function: encephalopathy or a patient suspected of having encephalopathy.
  - Electrolytes: serum sodium < 125 mEq/L, serum potassium < 3.3 mEq/L.
2. Highly likely to have abscess perforation due to malnutrition (albumin < 2.5 g/dL).
3. Symptoms of obstructive ileus.
4. Chronic anorexia, abdominal pain, and/or diarrhea symptoms before the onset of colorectal diverticulitis.
5. Performance status (an indicator of general condition and the degree of restriction of the patient’s daily life) ≥ 3 before the onset of colorectal diverticulitis.
6. History of DBT administration.
7. Treatment with insulin preparations.
8. Immunocompromised status.
9. Pregnancy or postpartum status.
10. Advanced allergy to Kampo formulas.

### Randomization, allocation concealment, and blinding

Baseline measures will be obtained before randomization. The web-based online randomization system used in this trial was provided by an independent academic research organization at Kyoto Prefectural University of Medicine. In this registration method, the principal investigator or research coordinator accesses the URL of the Web registration system and registers himself, and obtains the allocation result. For registered case information, the information entered on the case report form (CRF) form is faxed to the data center. Allocation of subjects to each treatment group is performed using the central registration system. In this registration method, researchers access THE Web-based registration and randomization system, and obtain the allocation. Participants are randomized using a minimization method, in which the history of diverticulitis (initial or recurrent) and the severity (with abscess formation on CT and/or C-reactive protein (CRP) ≧ 10 or without abscess formation and CRP < 10) are used as adjustment factor [11]. Enrolled patients who provide informed consent will be randomized 1:1 in a blinded manner into either an experimental treatment group receiving a 10-day DBT regimen plus conventional therapy or a control group receiving a 10-day placebo regimen plus conventional therapy. Only the allocation number is written on the test drug box, and only the data center knows whether the box contains DBT or the placebo. Thus, all participants and treatment providers, those measuring the outcomes, and those who are to analyze the data will be blinded to treatment allocation.

### Interventions

We will use DBT extract, which consists of five crude drugs in fixed proportions: *Rhei rhizoma* (2.0 g), *Natrium sulfuricum* (1.8 g), *Moutan cortex* (4.0 g), *Persicae semen* (4.0 g), and *Benincasae semen* (6.0 g), in 7.5 g of extract. DBT extract (Tsumura, Tokyo) - or placebo manufactured by Tsumura Co., Ltd., based on good manufacturing practice standards - at 2.5 g will be administered three times per day, 7.5 g per day, for 10 days. The placebo does not contain extract of DBT but was made following almost the same process, so that the shape is almost the same as for the DBT. In addition, in order to make the scent and taste closer to the actual drug, some fragrance that does not influence the effect was used. The administration of DBT or placebo begins immediately after informed consent and Web-based randomization. No restrictions will be imposed on the standard treatments for acute diverticulitis or any other disease while DBT is administered. If the clinical evolution is appropriate, a regular diet will be initiated, and the patient will be discharged; the patient will return as an outpatient 1 week later. We will ask all randomized patients whether they have relapsed, 1 year after discharge. When any serious adverse event occurs or patients do not wish to further participate in the trial, they will be excluded from the trial.

### Data collection

The study data collection process is outlined in Table 1.

**Table 1:**
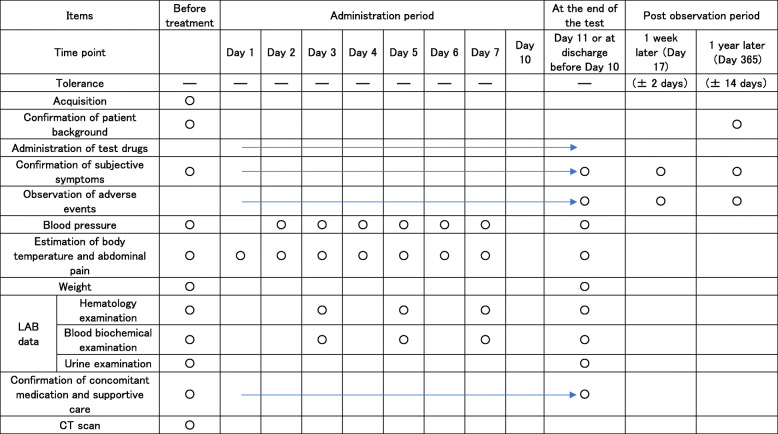
Treatment schedule and outcome measures

### Primary endpoint

Treatment success rate: If the following criteria are satisfied, treatment will be defined as successful:

1. In a patient with a fever and body temperature of 37.5 °C and abdominal pain requiring treatment at baseline, both fever relief within 3 days and elimination of abdominal pain within 4 days are confirmed.
2. In a patient with abdominal pain requiring treatment, but who did not have baseline fever and body temperature ≥ 37.5 °C, disappearance of abdominal pain is observed within 4 days.

The definition of symptom change is as follows:

- ① Fever relief
  Fever relief is defined as the absence of fever with body temperature ≥ 37.5 °C without the use of antipyretic analgesics. Even when an antipyretic analgesic is used, if the body temperature of 37.5 °C or more does not occur even after 6 h after the administration of the antipyretic analgesic, it is determined that the patient has fever relief.
- ② Elimination of abdominal pain
  Elimination of abdominal pain means that there is no abdominal pain without using antipyretic analgesics. Even when the antipyretic analgesic is used, it is determined that the abdominal pain has disappeared if no abdominal pain develops ≥ 6 h after the administration of the antipyretic analgesic. A visual analogue scale (VAS) score for pain should be obtained three times daily during the course of the abdominal pain.

### Secondary endpoints

The secondary endpoints are:

1. Number of hospital days: number of days until discharge, including the registration date, after trial registration.
2. Change in inflammatory response (CRP, WBC, neutrophil count): Differences between the value at the time of registration (baseline value) of each measurement and the value at each measurement day.
3. Thermal type: differences between the values at the time of registration (baseline value) of body temperature and the values on each measurement day.
4. Number of days until oral ingestion: number of days until the beginning of oral intake, including the registration date, after trial registration.
5. Recurrence rate (1-year follow-up observation): rate of recurrence at 1 year after randomization. Recurrence after discharge is regarded as diagnosis of colonic diverticulitis.
6. Adverse events (types and frequency of side effects): side effects will be recorded for each observed adverse event, and the type and frequency will be determined.

### Statistical consideration

#### Sample size

Calculation of the sample size in this trial was based on treatment success rate. The treatment success rate using the same criteria was estimated from the results of our retrospective study and it is 75% in the patients treated with DBT and 50% without DBT. In our previous study the dropout rate was 2%, therefore in our study we will assume that we will have a 2% dropout. We use the method proposed by Fleiss et al. Based on this, a sample size of 170 patients (85 per arm) is required to provide the trial with 90% power to detect the superiority of experimental treatment over control, at a two-sided alpha level of 5%. The sample size is also identical to the tables in Fleiss: *Statistical methods for rates and proportions* [12].

### Statistical analysis

The treatment success rate will be compared between groups using the Pearson chi-square test. Time to confirmed treatment-success will be summarized using the Kaplan-Meier method. Continuous variables will be summarized as mean and standard deviation, and comparisons will be made using the unpaired Student’s *t* test. Categorical variables will be summarized as frequency and proportions, and comparisons will be made using the chi-square test. Two-sided *p* values <0.05 will be considered statistically significant.

### Quality control and trial management

The management structure comprises the principle investigator (PI), a trial management group, and a data monitoring committee. The trial management group is responsible for conducting the trial and will meet monthly to discuss trial progress. The PI will visit each collaborative hospital for face-to-face meetings and sharing of information to promote patient recruitment. The data monitoring committee will review interim safety data and periodically review the conduct of the study. Additionally, all data will be monitored centrally every month. In this study, interim analysis of efficacy is not planned. Final analysis will be performed by the data center and our statistician. Additional monitoring may be performed at the discretion of the monitoring manager. The data monitoring committee has met once prior to the start of patient recruitment. At least twice per year, participating investigators, research assistants, and research nurses will be required to attend a training workshop for clinical research to ensure strict adherence to the study protocol and familiarity with the trial administration process. The data collected in this trial will comprise information recorded in case report forms and questionnaires. Data quality will be checked regularly by research assistants and overseen by monitors; all modifications will be marked on the case report forms, and data managers will recheck the data before they are officially logged. The database will be locked after all data have been cleaned. If participants withdraw from the trial during the study period, the reasons will be documented, and the dropout rate will be statistically analyzed.

## Discussion

The main purpose of this study is to assess the treatment effect of DBT in moderate to severe acute diverticulitis. The secondary aim of this study is to investigate the preventive effect of DBT in colonic diverticulitis. Patients with a history of diverticulitis are advised to consume a high-fiber diet or ingest fiber supplements, minimize red meat consumption, avoid non-aspirin non-steroidal anti-inflammatory drugs, stop smoking, maintain or achieve a healthy body weight, and exercise regularly [6]. Although multiple studies have investigated the methods of colonic diverticulitis prevention, the existing results are not supported by high-quality evidence. In this study, we will investigate differences between treatment with DBT combined with antibiotics, relative to treatment with placebo.

Diverticula can occur at any site in the colon. However, in Western countries they occur primarily in the sigmoid colon; in contrast, in Japan the right side of the colon or both sides of the colon are more commonly involved. The standard treatment for acute uncomplicated diverticulitis has been bowel rest (i.e., fasting), intravenous fluids, and intravenous antibiotics. There will be a diverse assortment of antibiotics used in this study, based on variability in the antibiotics used in clinical practice among the centers participating in the study. Additionally, while many trials have focused on surgical treatment of acute diverticulitis, there have been few studies dedicated to medical treatment of this condition. A review of the published data revealed that there is no standardization of the medical treatment of uncomplicated acute diverticulitis [13]. In this study, we will not limit the the types of antibiotics used; all antibiotics administered will be noted in the case report forms.

In Kampo medicine, internal abscesses generally comprise abscesses of the lungs and intestines. Similar to external abscesses, they are attributed to heat and constraint, which allow the formation of toxins and secondarily lead to the formation of pus. The appropriate strategy is to discharge the toxin, eliminate the phlegm, clear the heat, and open the constraint. If clumping of heat with stasis and stagnation is also present, herbs such as *Rhei Radix et Rhizoma* (*da hung*大黄) and *Moutan Cortex* (*mu dan pi*牡丹皮) can be added to drain heat and dispel stasis [8]. In this context, DBT is one of the most suitable formulas for treatment of acute diverticulitis. DBT might have preventive effects on the recurrence of diverticulitis, although the prognosis of patients was not reported in a prior study [9].

Most herbal medicine is orally administered, and many components of these medicines come into contact with the intestinal microflora in the alimentary tract. Some are modified by intestinal bacteria before their absorption into the bloodstream from the gastrointestinal tract [14]. Although administration of intravenous antibiotics may influence intestinal microflora and the effect of DBT might be altered, compared with classical therapy, our previous study showed that both intravenous antibiotics and DBT could be used at the same time [9].

DBT consists of five crude drugs: *Rhei rhizoma*, *Natrium sulfuricum, Moutan cortex, Persicae semen,* and *Benincasae semen*. Of these, *Rhei rhizoma* or rhubarb is one of the most important traditional herbal medicines, widely used in Kampo and traditional Chinese medicines for thousands of years, especially as a purgative. It might prevent bowel retention, overgrowth of toxic bacteria in patients with acute diverticulitis, and enable resolution of acute inflammation.

Studies of other functions of rhubarb in modern medical research in both clinical and basic science settings have revealed that rhubarb has multiple effects, including defervescence, anti-inflammatory actions, and expulsion of a variety of harmful materials (e.g., endogenous and exogenous toxins) from the bowel and the body.

It is shown that emodine in *Rhei rhizoma* protects against acute lung injury induced by lipopolysaccharide, and that its administration improves respiratory function [15]. These effects may be related to the reduction of inflammation in diverticulitis.

*Moutan cortex*, the root cortex of *Paeonia suffraticosa*, is an herbal medicine widely used as an analgesic, antispasmodic, and anti-inflammatory agent. *Moutan cortex* reportedly inhibits the secretions of interleukin-8, a major mediator of acute neutrophil-mediated inflammation, and macrophage chemoattractant protein-1, a potent mediator of chronic macrophage-mediated inflammation in human monocytic U937 cells [16]. Recently, *Moutan cortex* was shown to protect against sepsis induced by lipopolysaccharide/D-galactosamine [17]. The known chemical components of *Moutan cortex* include paeonol, paeonoside, paeonolide, paeoniflorin, benzoylpaeoniflorin, oxypaeoniflorin, benzoyloxy-paeoniflorin, and apiopaeonoside.

*Moutan cortex* inhibits the lipopolysaccharide plus interferon-γ induced expression of inducible nitric oxide synthase and tumor necrosis factor-α release. The lipopolysaccharide plus interferon-γ induced activation of nuclear factor-κB is almost entirely blocked by *Moutan cortex* [18].

*Persicae semen* or *Prunus persica*, is also well-known as a traditional medicine in Japan, China, and other Asian countries. It is frequently used as an ingredient in a variety of Kampo and Chinese medicine formulas, particularly those used to treat women’s diseases. The chemical constituents of the herb include cyanogenic glycosides, amygdalin, and prunasin as major components, along with glycerides, sterols, and emulsion. Amygdalin is abundant in the seeds of bitter almond and apricots of the *Prunus* genus, and other rosaceous plants. *Persicae semen* has anti-edema, anti-writhing, and anti-inflammation activities.

Each of these crude drugs has some anti-inflammatory effects, and the combination of these crude drugs may produce the synergy of Kampo medicine. Moreover, we are considering testing for correlation between the incidence of diverticulitis and body mass index (BMI), gender, age, and season, and between the success rate in treating diverticulitis and antibiotics, BMI, gender, age, and season.

In conclusion, this study protocol may contribute to the development of an effective treatment to help relieve acute diverticulitis. It may also provide evidence on the efficacy and safety of DBT in patients with acute diverticulitis.

### Trial status

Recruitment began in January 2018 and is expected to be completed in March 2020.

The latest protocol is Ver2.0, approved by the ethical committee on 25 July 2019.

## Supplementary information

**Additional file 1.** Standard Protocol Items: Recommendation for Interventional Trials (SPIRIT) 2013 checklist: recommended items to address in a clinical trial protocol and related documents

## Data Availability

The data will not be available for public access because of patient privacy concerns, but will be available from the corresponding author on reasonable request.

## Abbreviations

BMI: Body mass index
CRP: C-reactive protein
CT: Computed tomography
DBT: Daiobotanpito
WBC: White blood cells
